# Predictive value of admission D-dimer for contrast-induced acute kidney injury and poor outcomes after primary percutaneous coronary intervention

**DOI:** 10.1186/s12882-020-01743-7

**Published:** 2020-03-10

**Authors:** Kai-Yang Lin, Han-Chuan Chen, Hui Jiang, Sun-Ying Wang, Hong-Mei Chen, Zhi-Yong Wu, Feng Jiang, Yan-Song Guo, Peng-Li Zhu

**Affiliations:** 1 Department of Cardiology, Shengli Clinical Medical College of Fujian Medical University, Fujian Provincial Hospital, Fujian Provincial Center for Geriatrics, Fuzhou, 350001 China; 2 Department of Nursing, Fujian Provincial Hospital, Fujian Medical University, Fuzhou, 350001 China

**Keywords:** D-dimer, Contrast-induced acute kidney injury, Outcome, Primary percutaneous coronary intervention

## Abstract

**Background:**

DD was found to be associated with acute myocardial infarction (AMI) and renal insufficiency. However, it is uncertain whether DD is an independent risk factor of CI-AKI in patients undergoing pPCI.

**Methods:**

We prospectively enrolled 550 consecutive patients with STEMI undergoing pPCI between January 2012 and December 2016. The predictive value of admission DD for CI-AKI was assessed by receiver operating characteristic (ROC) and multivariable logistic regression analysis. CI-AKI was defined as an absolute serum creatinine increase ≥0.3 mg/dl or a relative increase in serum creatinine ≥50% within 48 h of contrast medium exposure.

**Results:**

Overall, the incidence of CI-AKI was 13.1%. The ROC analysis showed that the cutoff point of DD was 0.69 μg/ml for predicting CI-AKI with a sensitivity of 77.8% and a specificity of 57.3%. The predictive value of DD was similar to the Mehran score for CI-AKI (AUC_DD_ = 0.729 vs AUC_Mehran_ = 0.722; *p* = 0.8298). Multivariate logistic regression analysis indicated that DD > 0.69 μg/ml was an independent predictor of CI-AKI (odds ratio [OR] = 3.37,95% CI:1.80–6.33, *p* < 0.0001). Furthermore, DD > 0.69 μg/ml was associated with an increased risk of long-term mortality during a mean follow-up period of 16 months (hazard ratio = 3.41, 95%CI:1.4–8.03, *p* = 0.005).

**Conclusion:**

Admission DD > 0.69 μg/ml was a significant and independent predictor of CI-AKI and long-term mortality in patients undergoing pPCI.

## Background

Contrast-induced acute kidney injury (CI-AKI) is a common complication following coronary intervention procedures, which was associated with poor outcomes including dialysis, mortality, longer hospitalization and increased health-care costs [1–3], especially among patients undergoing primary percutaneous coronary intervention (pPCI) [4, 5]. Therefore, it is important to identify the patients who are likely to develop CI-AKI and take early preventive measures in this population.

D-dimer (DD) is a degradation product of cross-linked fibrin and a thrombosis marker, which is a well-established test and often used in the diagnosis and exclusion of pulmonary embolism and venous thromboembolism [6]. Ruptured plaque-induced thrombosis is a major cause of acute coronary syndrome (ACS) [7]. In recent years, DD has been reported to be a marker for the early diagnosis of ACS presenting with chest pain [8] and acute myocardial infarction (AMI) [9] . Furthermore, DD has been found to be associated with short-, mid- and long-term poor outcomes in patient with AMI [10–12]. In addition, as a marker of increased coagulability and fibrinolysis, DD also has been documented to be associated with renal insufficiency, which indicated that DD concentration increased significantly with increasing creatinine levels [13] or decreasing estimated glomerular filtration rate (GFR) [14]. However, none of the existing studies have established the relationship between DD levels and CI-AKI in patients after pPCI. Therefore, in a prospective single-center registry of patients undergoing pPCI, we sought to determine the predictive value of elevated admission DD levels for CI-AKI in patients with ST-elevation myocardial infarction (STEMI).

## Methods

### Study population

This is a prospective observational study conducted at Fujian Provincial Hospital, Fujian Cardiovascular Institute, from January 2012 to December 2016. A total of 639 consecutive patients with STEMI undergoing primary percutaneous coronary intervention (pPCI) were enrolled. Exclusion criteria were pregnancy, lactation, malignant tumor and < 1 year of life expectancy,end-stage renal disease (eGFR < 15 mL/min/1.73m^2^), long-term dialysis treatment, intra-vascular administration of contrast medium within the last 7 or 3 days postoperatively, lack of post-procedural serum creatinine (SCr) within 48 h after PCI,drug taking that decreasing kidney function 48 h before CM exposure,including sodium bicarbonate, non-steroidal anti-inflammatory drugs (NSAIDs), metformin, aminoglycoside drugs, cyclosporine, cisplatin and so on and severe valvular heart disease or preparation for other operations. Finally, 550 patients were included in the analysis. The study was approved by an institutional review committee and the subjects gave informed consent.

### Laboratory investigations, cardiac catheterization and medications

The serum concentration of DD was measured at admission for each patient before PCI. SCr was measured at admission and daily for the 2 days after contrast exposure. We also measured blood urea nitrogen (BUN), international normalized ratio (INR), fibrinogen, troponin I, N-terminal pro-B-type natriuretic peptide (NT-proBNP), white blood cell count, platelet, hemoglobin, and other parameters before PCI. PCI was performed by experienced interventional cardiologists according to standard clinical practice using standard guide catheters, guide wires, balloon catheters and stents via the femoral or radial approach. All patients received nonionic, low-osmolar contrast media (either Iopamiron or Ultravist, both 370 mg I/mL). In addition, 0.9% normal saline at a rate of 1 mL/kg/h was administered intravenously approximately 12 h during perioperative period (0.5 mL/kg/h if patients with heart failure). The use of medications includs antiplatelet agents (aspirin/clopidogrel), β-adrenergic blocking agents, statins, angiotensin-converting enzyme inhibitor (ACEI)/angiotensin receptor blocker (ARB), and other drugs which were at the discretion of the cardiologists according to clinical protocols based on interventional guidelines.

### Definitions and follow-up

The primary end point was CI-AKI, defined as an absolute SCr increase ≥0.3 mg/dl or a relative increase in serum creatinine ≥50% within 48 h of contrast medium exposure [15]. Additional end points included in-hospital outcomes [i.e.motality, recurrent myocardial infarction (MI), required renal replacement therapy (RRT),stent thrombosis, bleeding and length of hospital stay, hospital costs, and mortality] and long-term major adverse clinical events (MACEs). MACEs included mortality, stent restenosis, non-fatal myocardial infarction and target vessel revascularization (TVR).

The eGFR was calculated using the modified modification of diet in renal disease equation: 186.3 × SCr^-1.154^ × (age in years) ^-0.203^ × 1.212 (if patient was black) × 0.742 (if patient was female) [16].

Peri-hypotension was defined as systolic blood pressure (SBP) < 80 mmHg for at least 1 h requiring inotropic support with medications or intra-aortic balloon pump (IABP) within 24 h peri-procedure [17].

All patients were subject to follow-up for more than one year. Follow-up events were carefully monitored and recorded by trained nurses using either outpatient clinical visits or telephone contact with the patients or their relatives after discharge.

### Statistical analysis

All data were analyzed with SPSS version 20.0. We compared the baseline characteristics among 4 groups divided by DD quartiles. Normally distributed continuous variables are expressed as mean + standard deviation (SD). The Student’s t-test, Wilcoxon rank sum test or one way-analysis of variance was performed to determine the differences among groups. Categorical variables were compared by chi-square test or Fisher exact test. The receiver operating characteristic (ROC) curve was conducted to determine the cutoff value of DD and the Mehran score for CI-AKI.The difference between DD and the Mehran score for predicting CI-AKI was performed by nonparametric tests. Multivariate logistic regression analyses were performed to identify independent risk factors of CI-AKI. Cox regression analysis were performed to identify independent risk factors of long-term poor outcomes and the Kaplan-Meier curve was used to assess the survival time between different groups with log-rank test. A 2-sided *p* value <.05 was considered significant.

## Results

### Baseline characteristics

This study included 550 consecutive patients, of whom 72 (13.1%) developed CI-AKI.The mean age was 63.50 + 12.15 years and 67 (12.2%) were female. Baseline characteristics are described in Table 1, the patients were stratified into four DD quartiles: < 0.38μg/ml, 0.38–0.67μg/ml, 0.68–1.03μg/ml, and > 1.03μg/ml. Patients in the higher DD group were significantly older, more likely to have anemia and worse renal function, had higher baseline of NT-proBNP, cholesterol, low density lipoprotein-cholesterol (LDL-C), fibrinogen, INR, Mehran score, and had a higher percentage of perioperative hypotension, use of contrast volume and IABP, but lower diastolic blood pressure and left ventricular ejection fraction (LVEF). And baseline vascular acess between CI-AKI group and Non-CIAKI group are described in Supplement Table 1.

**Table 1:** Baseline Characteristics Among the 4 Groups Divided by DD Quartiles

Variables	Q1 (*n* = 141) (< 0.38)	Q2 (*n* = 140) (0.38–0.67)	Q3 (*n* = 135) (0.68–1.03)	Q4 (*n* = 134) (> 1.03)	*p*
Demographics					
Age, years	59.79 ± 11.54	61.48 ± 12.46	64.51 ± 11.17	68.48 ± 11.68	< 0.0001
Age > 75 years, n (%)	9 (6.4%)	16 (11.4%)	25 (18.5%)	45 (33.6%)	< 0.0001
Sex, female, n (%)	10 (7.1%)	14 (10.0%)	22 (16.3%)	21 (15.7%)	0.053
Systolic blood pressure,mmHg	122.39 ± 23.44	123.73 ± 22.25	121.80 ± 22.66	116.52 ± 23.86	0.123
Diastolic blood pressure,mmHg	74.84 ± 17.49	75.10 ± 15.98	72.89 ± 16.38	68.37 ± 15.56	0.012
Heart rate,bpm	77.06 ± 12.07	79.16 ± 14.85	77.92 ± 15.47	77.98 ± 16.23	0.795
Medical history					
Previous PCI, n (%)	3 (2.1%)	5 (3.6%)	5 (3.7%)	5 (3.7%)	0.851
Smoker, n (%)	85 (60.3%)	86 (61.4%)	76 (56.3%)	79 (59.0%)	0.842
Diabetes, n (%)	35 (24.8%)	30 (21.4%)	35 (25.9%)	40 (29.9%)	0.456
Hypertension, n (%)	78 (55.3%)	81 (57.9%)	79 (58.5%)	82 (61.2%)	0.804
Anemia, n (%)	26 (18.4%)	36 (25.7%)	33 (24.4%)	55 (41.0%)	< 0.0001
Prior MI, n(%)	3 (2.1%)	3 (2.1%)	4 (3.0%)	1 (0.7%)	0.628
Laboratory measurements					
Serum creatinine, mg/dl	0.82 ± 0.18	0.82 ± 0.19	0.86 ± 0.30	1.00 ± 0.41	< 0.0001
Serum creatinine > 1.5 mg/dl, n	0 (0.0%)	0 (0.0%)	6 (4.4%)	13 (9.7%)	< 0.0001
eGFR<60 mL/min/1.73m^2^, n(%)	3 (2.1%)	3 (2.1%)	13 (9.6%)	23 (17.2%)	< 0.0001
Hemoglobin, g/L	143.04 ± 13.28	141.66 ± 15.08	140.81 ± 14.76	136.04 ± 19.99	0.002
Hematocrit	0.41 ± 0.04	0.41 ± 0.04	0.41 ± 0.04	0.40 ± 0.06	0.008
Cholesterol, mmol/L	5.00 ± 1.08	4.95 ± 1.24	4.75 ± 1.09	4.58 ± 1.10	0.007
LDL-C, mmol/L	3.40 ± 0.99	3.39 ± 1.06	3.21 ± 0.98	3.02 ± 1.00	0.007
Triglycerides, mmol/L	1.70 ± 1.25	1.48 ± 1.01	1.39 ± 0.93	1.44 ± 1.33	0.103
LgNT-pro-BNP, pg/mL	2.26 ± 0.60	2.26 ± 0.63	2.40 ± 0.59	2.60 ± 0.75	< 0.0001
Fibrinogen, g/L	3.44 ± 0.75	3.51 ± 0.97	3.52 ± 0.92	3.78 ± 1.06	0.020
INR	1.08 ± 0.10	1.09 ± 0.10	1.15 ± 0.30	1.16 ± 0.14	< 0.0001
DD,ug/ml	0.25 ± 0.08	0.52 ± 0.09	0.85 ± 0.10	1.71 ± 1.42	< 0.0001
LVEF	56.17 ± 7.57	54.08 ± 7.31	53.91 ± 7.24	49.63 ± 9.67	< 0.0001
LVEF < 0.45, n(%)	9 (6.4%)	17 (12.1%)	17 (12.6%)	42 (31.3%)	< 0.0001
Medication					
Statin use, n (%)	140 (99.3%)	140 (100.0%)	135 (100.0%)	132 (98.5%)	0.285
Antiplatelet agents use, n (%)	141 (100.0%)	140 (100.0%)	135 (100%)	132 (98.5%)	0.101
Procedural characteristic					
Perioperative IABP, n (%)	0 (0.0%)	2 (1.4%)	3 (2.2%)	8 (6.0%)	0.009
Perioperative hypotension, n (%)	47 (33.3%)	42 (30.0%)	49 (36.3%)	62 (46.3%)	0.034
Contrast volume, ml	167.80 ± 53.57	181.79 ± 50.74	187.19 ± 51.93	189.18 ± 53.76	0.003
Contrast volume ≥ 150 ml, n (%)	79 (56.0%)	100 (71.4%)	100 (74.1%)	103 (76.9%)	0.001
Number of stents	1.34 ± 0.54	1.33 ± 0.55	1.33 ± 0.52	1.18 ± 0.42	0.116
Number of diseased vessels, n	2.16 ± 0.90	2.18 ± 0.86	2.41 ± 0.81	2.23 ± 0.83	0.147
Stent length,mm	36.62 ± 17.54	35.10 ± 17.52	36.92 ± 18.39	31.75 ± 12.17	0.134
Merhan risk score	7.50 ± 4.99	8.04 ± 4.50	8.97 ± 5.48	11.64 ± 5.79	< 0.0001
Vascular access					0.622
Radial access	125 (88.7%)	120 (85.7%)	121 (89.6%)	117 (87.3%)	
Femoral accessra	16 (11.3%)	17 (12.2%)	11 (8.2%)	14 (10.5%)	
Radial access + femoral access	0 (0.0%)	3 (2.1%)	3 (2.2%)	3 (2.2%)	

Abbreviations: *DD* D-Dimer; *PCI* percutaneous coronary intervention, *MI* myocardial infarction, *LVEF* left ventricular ejection fraction, *eGFR* estimated glomerular filtration rate, *LDL-C* low density lipoprotein-cholesterol, *NT-pro-BNP* N-terminal pro-B-type natriuretic peptide, *INR* international normalized ratio, *IABP* intra-aortic balloon pump

### DD level predicts CI-AKI by ROC curve

**Fig. 1:**
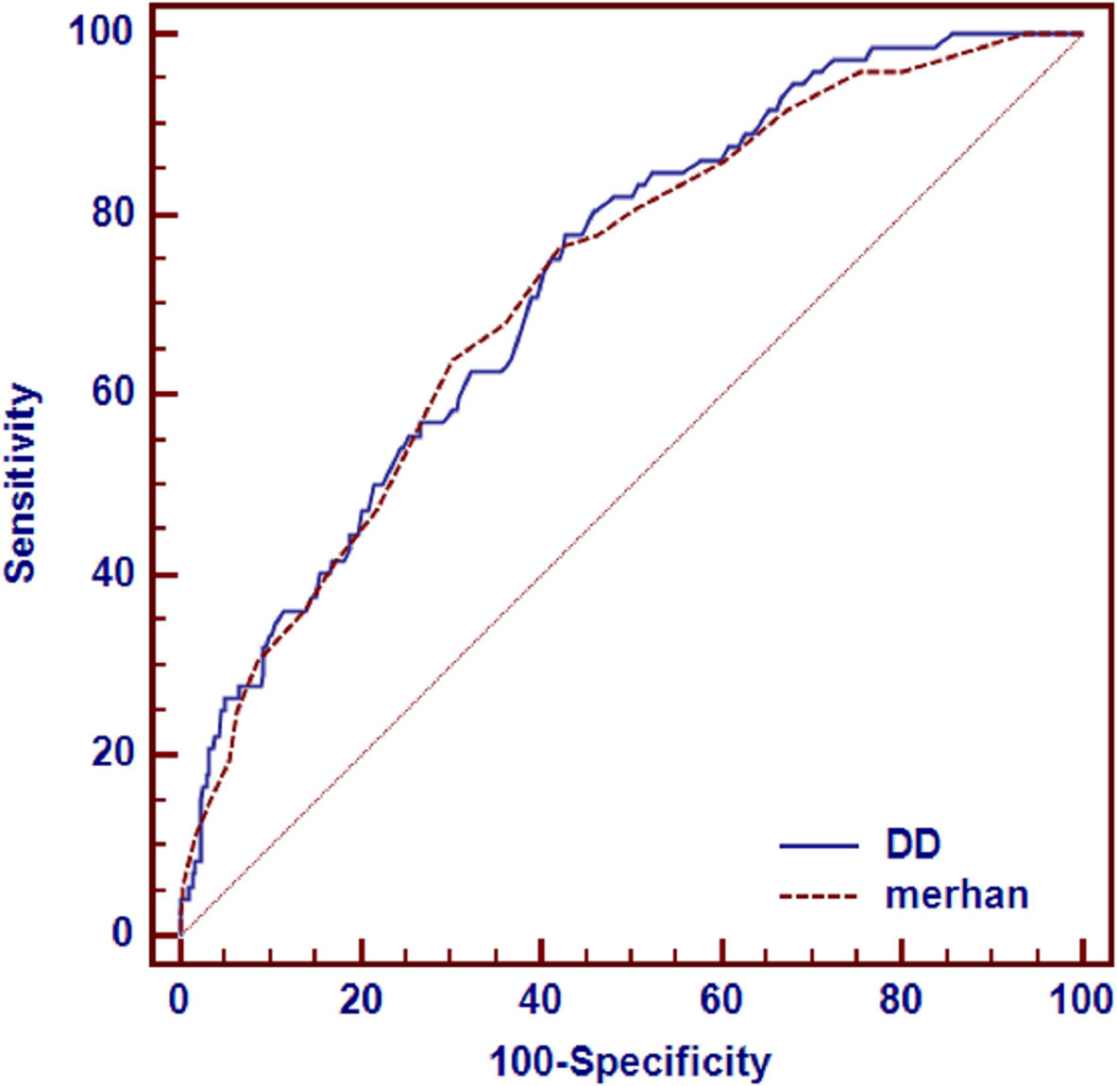
ROC curves of DD and Mehran score for CI-AKI

A DD cutoff point of 0.69μg/ml predicted by the ROC curve had a sensitivity of 77.8% and a specificity of 57.3%(AUC = 0.729, 95% confidence interval [CI]: 0.690–0.766, *p* < 0.0001; Fig. 1). The predictive value of DD was similar to the Mehran score for CI-AKI (AUC_DD_ = 0.729 vs AUC_Mehran_ = 0.722, *p* = 0.8298).(Fig. 1).

### In-hospital, 6-months and 1-year adverse events

The incidences of CI-AKI, in-hospital mortality and required RRT were significantly higher in patients with DD > 0.69μg/ml than DD ≤ 0.69μg/ml(23.0% vs 5.2%,7.4% vs 1.0, 2.9% vs 0.3%, all *p* < 0.05). Patients with DD > 0.69μg/m also had significantly higher 6-month and 12-month mortality (9.0% vs 1.0, 9.8% vs 1.6%, both *p* < .05) and much higher hospital costs (76,070.04 ± 43,063.55 vs 66,165.01 ± 28,815.73 renminbi; *p* < .01).

Furthermore, CI-AKI was related to higher risk of in-hospital mortality (16.7% vs 1.9%, *p* < .001), required RRT, bleeding(16.7% vs 1.9,5.6% vs 0.8, 5.6% vs 0.8%, all *p* < .05), and significantly increased hospital stay length and hospital costss (15.21 ± 10.30 vs 12.75 ± 6.42, 88,876.34 ± 56,971.9 vs 67,989.403 ± 1357.43 renminbi; *p* < .01). (Table 2).

**Table 2:** Incidence of In-Hospital Outcomes

Outcome	DD ≤ 0.69(*n* = 306)	DD > 0.69 (*n* = 244)	*P*-value	CI-AKI(−) (*n* = 478)	CI-AKI(+) (*n* = 72)	*P*-value
CI-AKI	16 (5.2%)	56 (23.0%)	< 0.0001			
In-hospital mortality, n (%)	3 (1.0%)	18 (7.4%)	< 0.0001	9 (1.9%)	12 (16.7%)	< 0.0001
Recurrent MI, n (%)	1 (0.3%)	4 (1.6%)	0.176	4 (0.8%)	1 (1.4%)	0.506
Required RRT, n (%)	1 (0.3%)	7 (2.9%)	0.025	4 (0.8%)	4 (5.6%)	0.013
Stent thrombosis, n (%)	1 (0.3%)	1 (0.4%)	1.000	1 (0.2%)	1 (1.4%)	0.245
Bleeding, n (%)	4 (1.3%)	4 (1.6%)	0.738	4 (0.8%)	4 (5.6%)	0.013
Hospitalization length, days	12.52 ± 6.49	13.72 ± 7.68	0.081	12.75 ± 6.42	15.21 ± 10.30	0.015
Hospital costs, RMB	66,165.01 ± 28,815.73	76,070.04 ± 43,063.55	0.005	67,989.403 ± 1357.43	88,876.34 ± 56,971.9	< 0.0001
6-months mortality	3 (1.0%)	22 (9.0%)	< 0.0001	13 (2.7%)	12 (16.7%)	< 0.0001
12-months mortality	5 (1.6%)	24 (9.8%)	< 0.0001	17 (3.6%)	12 (16.7%)	< 0.0001

Abbreviations: *CI-AKI* contrast-induced acute kidney injury, *DD* D-Dimer, *RRT* renal replacement therapy, *MI* mycardial infarction, *RMB* renminbi

### Risk factors of CI-AKI

**Fig. 2:**
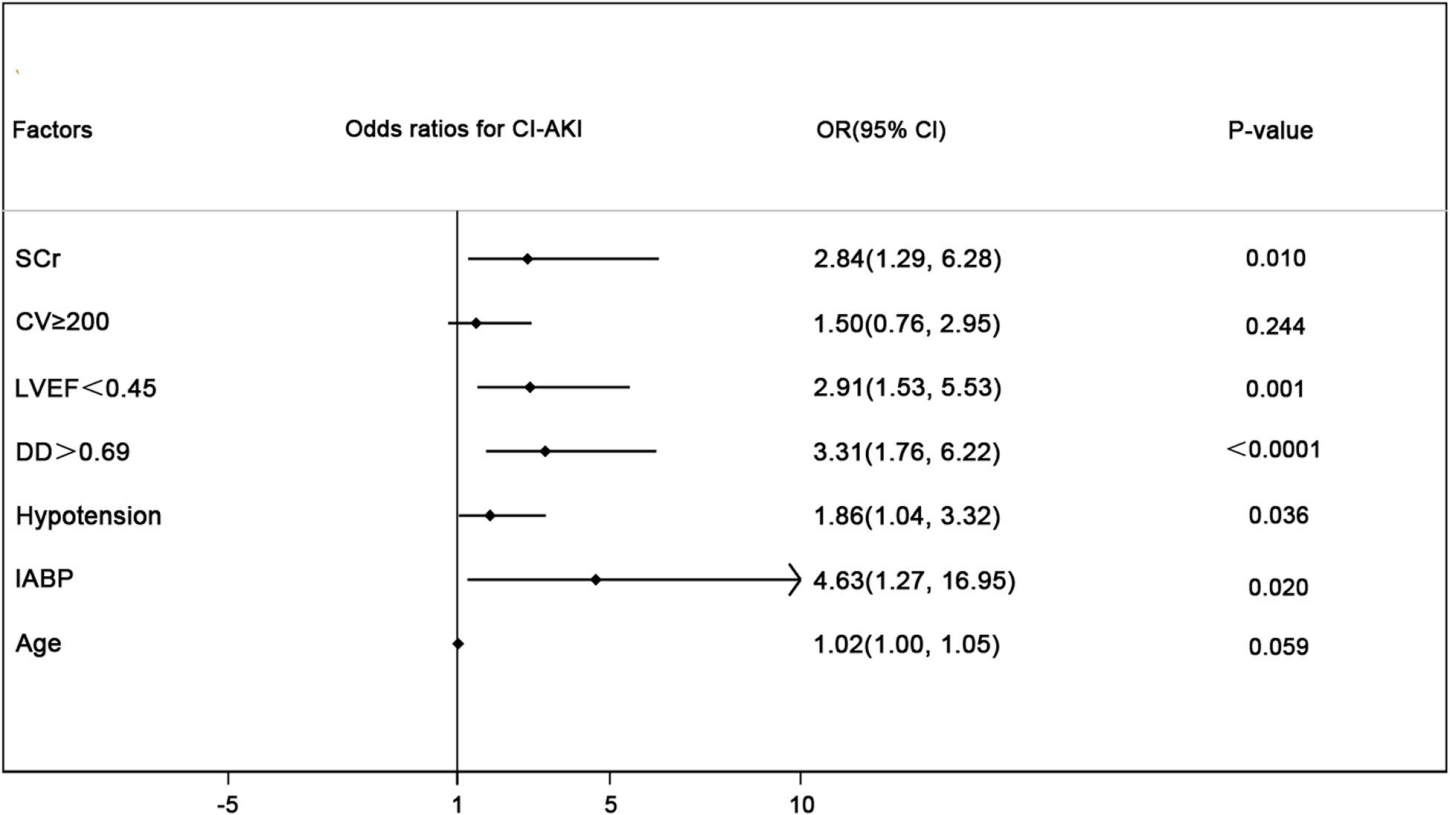
Multivariate logistic analysis for CI-AKI

Univariate logistic regression analysis indicated that the contrast volume ≥ 200 ml, LVEF < .45, SCr, perioperative hypotension, use of IABP, and DD > 0.69μg/ml were significantly associated with CI-AKI after pPCI (all *p* < .05). After adjusting for potential confounding risk factors, LVEF < .45(adjusted odds ratio [OR] 2.79, 95% CI 1.47–5.28, *p* = .002), SCr (OR 2.84, 95% CI 1.29–6.28, *p* = .010), perioperative hypotension (OR 2.03, 95% CI 1.13–3.64, *p* = .017), use of IABP (OR 4.55, 95% CI 1.27–16.34, *p* = .020) and DD > 0.69μg/ml (OR 3.37, 95% CI 1.80–6.33, *p* < .0001) remained significant predictors of CI-AKI. (Fig. 2).

### DD level and long-term outcomes

**Fig. 3:**
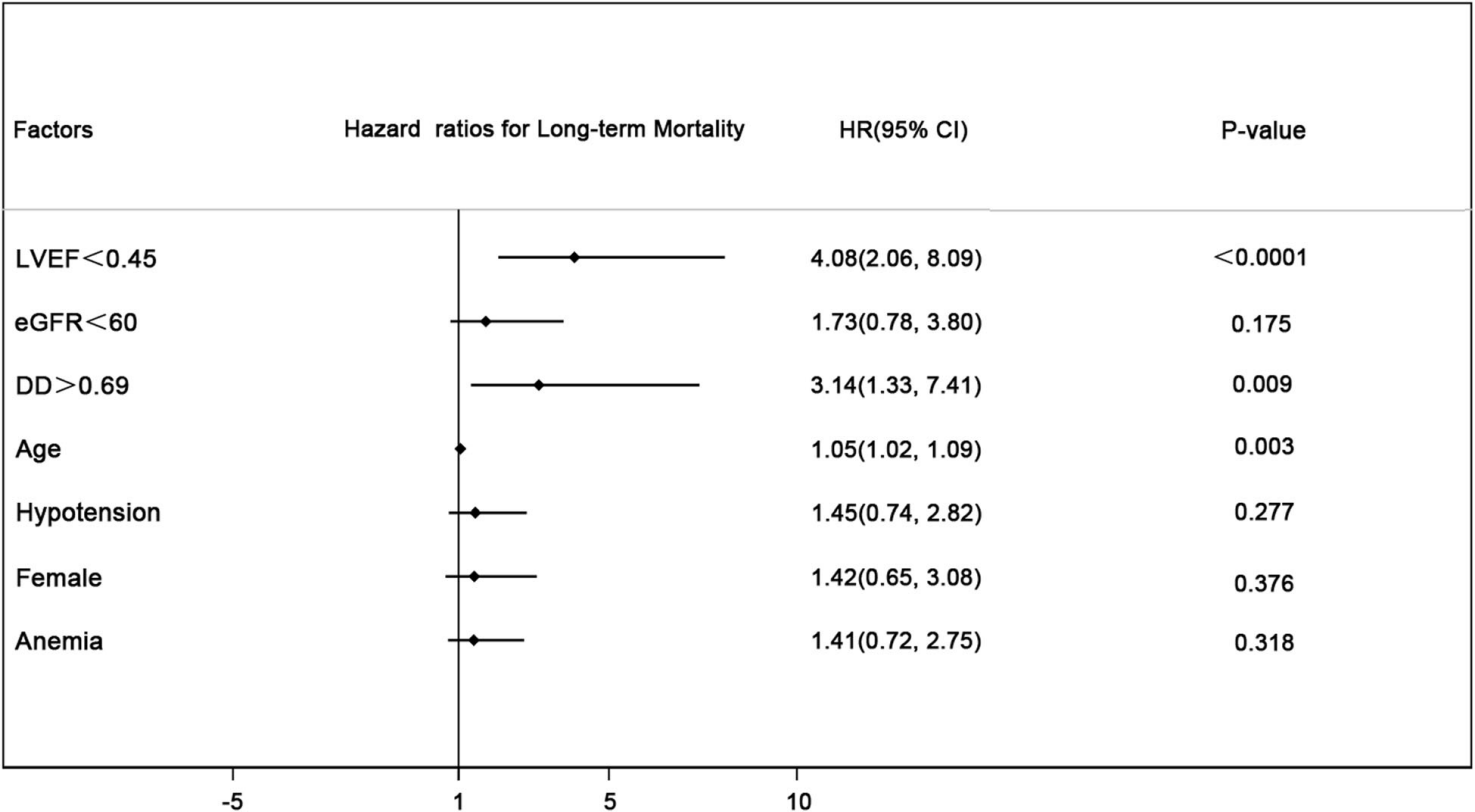
Cox regression analysis for long-term mortality

The median follow-up period was 16 months. Cox regression analysis revealed that DD > 0.69 μg/ml was an independent risk factor for long-term mortality (hazard ratio [HR] = 3.41, 95%CI:1.4–8.03, *p* = .005) after adjusting for other risk factors including LVEF < .45, eGFR<60 mL/min/1.73m^2^, perioperative hypotension, female, anemia. (Fig. 3).

**Fig. 4:**
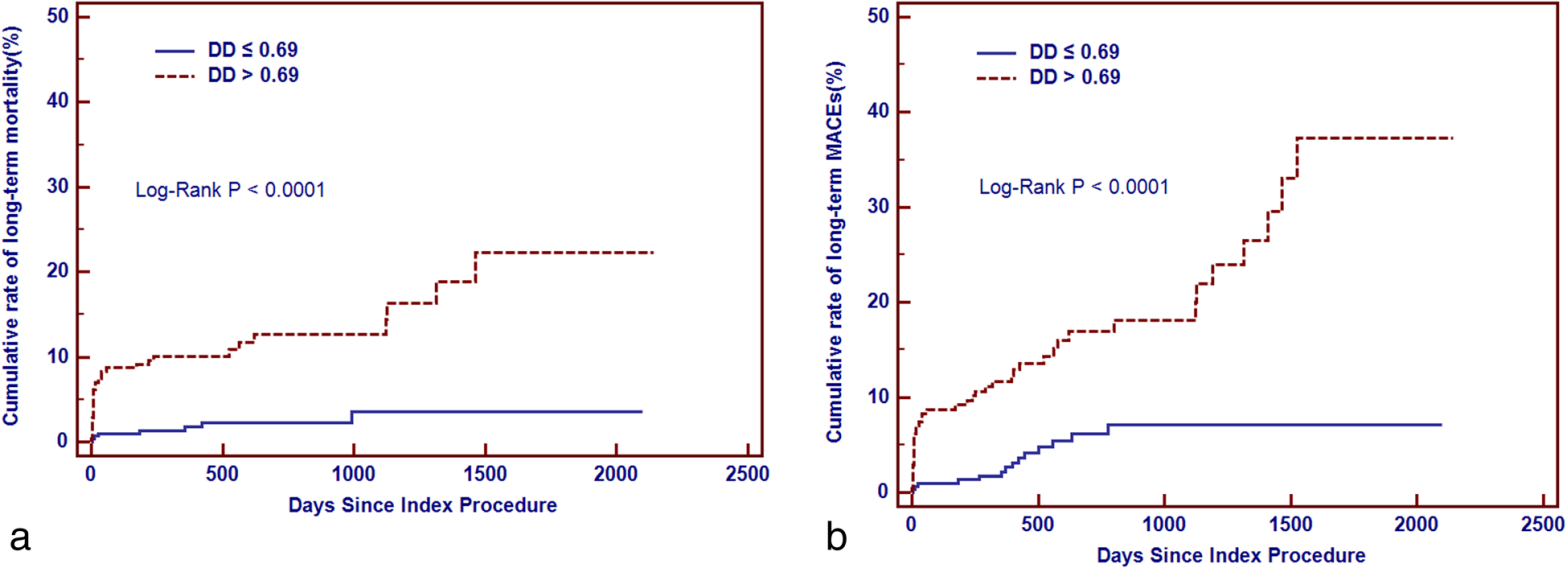
**a** Cumulative rate of mortality between patients with low DD and high DD level. **b** Cumulative rate of MACEs between patients with low DD and high DD level

**Fig. 5:**
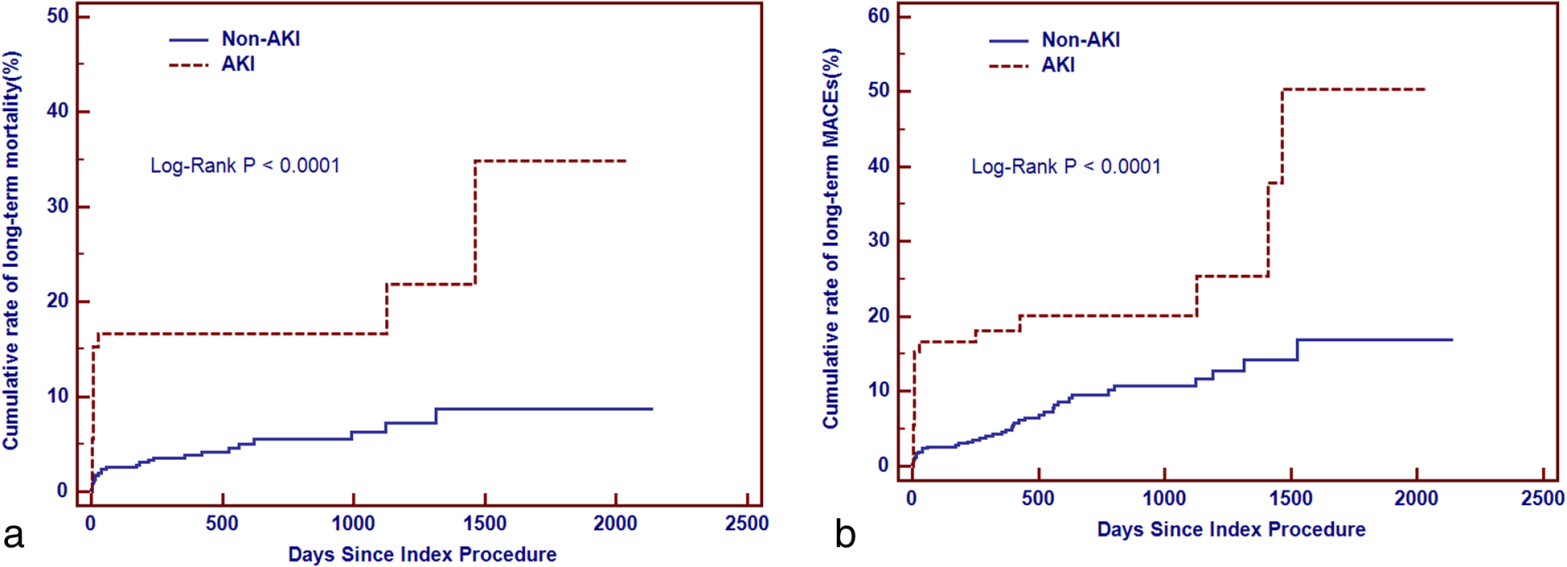
**a** Cumulative rate of mortality between patients with AKI and non-AKI. **b** Cumulative rate of MACEs between patients with AKI and non-AKI

Compared with patients with DD ≤ 0.69μg/ml, the Kaplan-Meier curve showed that patients with DD > 0.69 μg/ml had higher rate of all-cause mortality and MACEs (Chi-Square = 22.93, Log-Rank *p* < 0.0001; Chi-Square = 24.16, Log-Rank *p* < 0.0001,respectively).(Fig. 4 A-B) Patients who developed CI-AKI had a higher rate of all-cause mortality and MACEs compared with those who without (Chi-Square = 20.12, Log-Rank *p* < 0.0001; Chi-Square = 16.24, Log-Rank *p* < 0.0001,respectively).(Fig. 5 A-B).

## Discussion

To our knowledge, this study is the first to investigate the relationship between DD and CI-AKI. The main finding of our study was that the elevation of admission DD was markedly related to the incidence of CI-AKI. DD > 0.69 μg/L was found to be the best cutoff point for predicting CI-AKI with a sensitivity of 77.8% and a specificity of 57.3% after pPCI, which exhibited similar predict value to the Mehran score. Furthermore, DD > 0.69 μg/L was also strongly associated with in-hospital and long-term mortality in this population.

Previous studies had demonstrated that an increased risk of CI-AKI in patients undergoing pPCI compare with elective PCI [18–20], as the patients who underwent pPCI usually accompanied by hemodynamic instability, severe heart failure, and insufficient hydration. In our study, the incidence of CI-AKI in patients undergoing pPCI was up to 13.1%, which was consistent with the data available in the literature [21, 22].

Multiple risk factors have been confirmed to be associated with CI-AKI and several risk models have been applied to predict CI-AKI in clinical practice. Merhan score is the most common tool for using in the assessment of the risk of CI-AKI. However, the score need 8 periprocedural risk factors including hypotension, intra-aortic balloon pump, congestive heart failure, chronic kidney disease, diabetes, age > 75 years, anemia, and volume of contrast, some of which are not available before pPCI [17]. In our study, we also have found LVEF < 0.45, SCr, perihypotension,use of IABP were independent risk factors for prediciting CI-AKI, as well as DD may be a new biomarker for predicting CI-AKI in patients undergoing pPCI, which was not significantly different from the Merhan CI-AKI score (*P* = 0.410). In addition, the DD test is routinely performed at admission in our department for patients with cardivoscular disease. Therefore, compared with Mehran score, DD may be a more easily and timely available tool for identifying high-risk patients of CI-AKI in STEMI patients undergoing pPCI.

DD is a plasmin-derived soluble degradation product of cross-linked fibrin, which could provide a rapid assessment of thrombotic activity and the severity of thrombotic burden [23], reflecting the diagnosis and prognosis of many disorders. DD has been already widely accepted in diagnosis of venous thromboembolism and pulmonary embolism which is sensitive but not well specific [24–26]. Moreover, numerous studies found that DD testing was also valuable in other disease, especially cardiovascular disease such as acute aortic dissection [27], atrial fibrillation [28], heart failure [29, 30] and so on.

Previous studies also found that elevated DD level was a useful biomarker for kidney disease. Michael G. Shlipak et al. evaluated a population-based cohort study of 5888 subjects aged ≥65 years from Cardiovascular Health Study, and showed that [31] DD levels were significantly higher among patients with renal insufficiency(*P* = 0.0002) and increased as renal function declined regardless of cardiovascular disease status(*p* = 0.004). The conclusion was similar to the study by Wannamethee, S. G et al., which indicated that activated coagulation marker (DD) increased with decreasing eGFR in elderly men [32]. Spring JL also demonstrated that [13] there was a statistically significant correlation between DD concentration and renal function in the critically ill patients. Another recent study [14] found that in patients undergoing cardiac surgery, prothrombin fragment 1.2(coagulation marker) levels were significantly higher in the AKI group (506 ± 548 vs.999 ± 704.1 pmol/L;*p* = 0.018) and they were independently associated with eGFR reduction, with an area under the ROC of 0.744. The association between the degree of coagulation’s activation and AKI following cardiac operations may be partly explained by microcirculation impairment and dysfunction of endothelial cells. As were described above, DD were reported to be an important biomarker in patients with renal insufficiency or AKI. However, to our knowledge, there was not even a study focusing on the relationship between DD levels and CI-AKI. Now our results fill in this gap by documented that elevated DD level was a strong and independent predictor of CI-AKI after pPCI and CI-AKI, even after adjusting for potential confounding factors. Meanwhile, a pre-procedural DD levels>0.69 μg/ml was found to be a best cutoff point for predicting the risk of CI-AKI, with 77.8% sensitivity and 57.3% specificity, which exhibited similar predictive value to the Mehran score.

The mechanisms underlying the association between DD levels and CI-AKI is uncertain. The potential pathophysiological mechanism may be partly explained by following. First, DD levels were confirmed to be closely related to the occurrence of no-reflow after pPCI [33] indicating that the thrombus is unstable and easily to fall off into the circulation system, which may influence the renal blood flow and further lead to CI-AKI. Second, elevated DD levels is mainly cleared through renal excretion, so higher DD levels often reflect the injury of renal function, which indirectly revealing the decline in the kidney’s ability to excrete the contrast agent, further to enhance the direct cytotoxicity of contrast agent. Third, DD levels also related to other well-known risk factors for CI-AKI, such as advanced age [34], heart failure [29, 30]. However, after adjusting above potential influence factors on DD, elevated admission DD levels remained a powerful risk factor of CI-AKI, which indicated that additional pathophysiological processes might play a role on DD in STEMI patients after pPCI DD. Finally, some studies [35, 36] found that prothrombotic states may active inflammatory reaction through inducing the release of IL-1β, IL6, P-selectin and so on,which may also be involved in the process of CI-AKI.

In addition, DD also was found to be potentially useful in diagnosis and prognosis in patients with AMI. Elevated serum levels of DD were first time discovered in acute transmural myocardial infarction patients after thrombolytic therapy with intravenous streptokmase in 1986 [37]. Thereafter, several evidence revealed that the diagnostic and prognostic value of elevated DD in patients with AMI. A small, single-center study of 257 patients with acute chest pain by Bayes-Genis A et al. [8] found that DD level > 500 microg/L had an independent early diagnostic value for MI and increased the diagnostic sensitivity of the electrocardiogram and history from 73 to 92%. Furthermore, DD was found to be associated with poor outcomes in patients with AMI. Akgul O et al. [10] demonstrated that a high admission DD level (> 0.72 μg/ml FEU) was a powerful independent predictor of 6-month all-cause mortality. Another study of HORIZONS-AMI trial [12] showed admission DD levels were a strong predictor of MACE in patients treated with pPCI within 3 years follow up. Compared with patients with lower DD levels(< 0.71 μg/ml), patients with higher DD levels(≥0.71 μg/ml) on admission were associated with an adjusted hazard ratio of 2.58 for MACE. In our study, poor prognosis for short- and long-term mortality in patients undergoing pPCI having higher DD levels were also observed. Compared with patients with lower DD levels(≤0.69 μg/ml), patients with higher DD levels(>0.69 μg/ml) had significantly higher incidences of in-hospital outcomes such as CI-AKI, mortality and required RRT,and increased hospital costs, as well as higher 6-month and 12-month mortality. Furthermore, DD levels>0.69 μg/ml was also associated with an increased risk of all-cause mortality during 2 years of follow-up.

### Limitations

We acknowledge several limitations in our study. First, this study was a single-center, observational study and may be affected by confounding and selection biases. However, we were careful to include consecutive patients. Second, data about peri-procedural hydration volume which may influence the incidence of CI-AKI was not recorded. Third, variations in the time to measurement may have led to missed peak levels of creatinine after the procedure,further to cause an underestimation of the true incidence of CI-AKI. Fourth, we did not analyze the relationship between DD level and the high-sensitivity C-reactive protein,other proinflammatory cytokines and the incidence of no-reflow in detail,the defined mechanism of DD-induced CI-AKI is still unclear,which should be evaluated in a future trial. Fifth, we just found an indicator may be related to the occurrence of CI-AKI, whose relationship is causal or accidental remains unclear. Sixth,the consensus definition of CI-AKI may be a potential confounder when considering the specific forms of AKI which may occur in variable timeframes. Although we tried to exclude other risk factors for renal failure,it is difficult to rule out the patients developing renal failure following contrast media exposure but not having contrast nephropathy completely. Seventh, the long term kidney function was limited in our study. Despite these limitations, our results provided useful insights into the correlation of serum DD levels with the incidence of CI-AKI.

## Conclusions

Our study found that admission elevated DD level may be markedly related to an increased risk of CI-AKI in patients undergoing pPCI. In addition, elevated DD level is also found to be associated with short- and long-term poor outcomes. DD is a simple, rapid, cheap laboratory test that can be performed before pPCI to identity the risk of CI-AKI in patients with STEMI. Patients at high risk of CI-AKI with admission elevated DD level should be paid attention to take preventive measures during the perioperative period.

## Supplementary information

**Additional file 1: Table S1.** Baseline Vascular Access between CI-AKI Group and Non-CIAKI Group.

## Data Availability

The datasets used and/or analysed during the current study are available from the corresponding author on reasonable request.

## Abbreviations

BUN: Blood urea nitrogen
CI-AKI: Contrast-induced Acute Kidney Injury
DD: D-dimer
eGFR: Estimated glomerular filtration rate
IABP: Intra-aortic balloon pump
LDL-C: Low density lipoprotein-cholesterol
LVEF: Left ventricular ejection fraction
pPCI: Primary percutaneous coronary intervention
SCr: Serum creatinine
