# Supplementary material for: Predictive value of admission D-dimer for contrast-induced acute kidney injury and poor outcomes after primary percutaneous coronary intervention
Source: BMC Nephrol. 2020 Mar 10;21:90. doi: 10.1186/s12882-020-01743-7 (PMC7063708; doi:10.1186/s12882-020-01743-7)
Supplement: Supplementary file 1 — Additional file 1: Table S1. Baseline Vascular Access between CI-AKI Group and Non-CIAKI Group. [file 12882_2020_1743_MOESM1_ESM.docx]

**Supplement Table1 Baseline Vascular Access between CIAKI Group and Non-CIAKI Group**

| Variables | Total  （n=550） | Non- CIAKI（n=478） | CIAKI（n=72） | *P*-value |
| --- | --- | --- | --- | --- |
| **Vascular access** |  |  |  | 0.422 |
| Radial access | 478(86.9%) | 423(88.5%) | 60(83.3%) |  |
| Femoral access | 58(10.5%) | 48(10.0%) | 10(13.9%) |  |
| Radial access + femoral access | 9(1.6%) | 7(1.5%) | 2(2.8%) |  |
